# Is food insecurity contributing to malnutrition in older adults in Switzerland? – A cross-sectional study

**DOI:** 10.3389/fnut.2023.1228826

**Published:** 2023-08-16

**Authors:** Maurus Rigling, Philipp Schuetz, Nina Kaegi-Braun

**Affiliations:** ^1^Division of General Internal and Emergency Medicine, Medical University Department, Kantonsspital Aarau, Aarau, Switzerland; ^2^Medical Faculty, University of Basel, Basel, Switzerland

**Keywords:** food security, malnutrition, health, quality of life, older adults, food insecurity, nutritional risk, risk factors

## Abstract

**Background:**

Food insecurity has been defined as “*limited access to food, at the level of individuals or households, due to lack of money or other resources*” and may increase the nutritional risk, which in turn leads to poor health, development of chronic diseases, poor psychological and cognitive functioning, and substandard academic achievements. There is limited data on the importance of food insecurity in a rich country such as Switzerland.

**Methods:**

This is a cross-sectional analysis of data from a structured survey in an elderly population of Switzerland. The data was assessed between June and August 2021 in the course of a 7-year phone call follow-up from the EFFORT trial, which included medical inpatients at nutritional risk from 2014 to 2018. A validated questionnaire (Six-Item Short Form 2012 of the U.S: Household Food Security Survey Module) was used to assess food security status.

**Results:**

Of the 433 included patients, 30 (6.9%) were food insecure. A significant association between food insecurity and age, governmental financial support and self-reported loneliness was found. When compared with the food secure group, there was a significant lower quality of life measured by the EQ-5D VAS.

**Conclusion:**

In an older Swiss population of patients at nutritional risk, food insecurity was named as a contributing factor for malnutrition in about 7% of patients, particularly younger individuals with financial support, and self-reported loneliness. In the assessment of malnutrition, physician and dieticians should ask for food insecurity and if detect take appropriate actions.

## 1. Introduction

Since 2014 the global prevalence of moderate or severe food insecurity has been slowly on the rise (1). Food insecurity has been defined as “*limited access to food, at the level of individuals or households, due to lack of money or other resources*” and has been associated with poor dietary intake and nutritional status, poor health, increased risk for the development of chronic diseases, poor psychological and cognitive functioning, and substandard academic achievement (2).

In 2020 it was estimated that the increase of food insecurity was equal to that of the previous 5 years combined (1). Worldwide around 2.4 billion people suffer from some form of food insecurity in Africa and Asia (1). On a much smaller scale, food insecurity is also a problem in high-income countries (3–6). In Europe and Northern America, studies found that in 2020 around 9 % of the population were moderately or severely food insecure (1). In these countries, food insecurity can coexist with food waste, overproduction and abundant food availability (7). In recent years, this was especially the case in countries, that suffered a financial crisis (8, 9), while currently the COVID-19 pandemic globally further increased the risk for food insecurity (10, 11). The consequences of food insecurity are most visible in low-income countries, where hunger-related malnutrition is a serious problem. In high-income countries, undernutrition is more commonly seen in ill patients (disease-related malnutrition, DRM) resulting from anorexia, catabolic metabolism and immobility. According to a recent meta-analysis of Kantilafti et al., there is a reverse relationship between food insecurity and multimorbidity (12). They found a 1.5-fold increased probability of multimorbidity among people with food insecurity. Conversely, people with multimorbidity had more than two times higher odds to present with food insecurity. Food security, morbidity and malnutrition are therefore supposed to have a complex interplay. Despite the rising prevalence of food insecurity and its burden on health, there is a lack of evidence in many European countries, including Switzerland (4). While in the United States and Canada food security is routinely monitored, there is no such monitoring in Switzerland and consequently data on food insecurity are missing (13, 14).

Even though Switzerland has a high standard of living, a low poverty rate and strong welfare programs, the question about the existence of food insecurity should still be raised. We hypothesize that there is a relevant amount of food insecure people living in Switzerland, especially in the time of the COVID-19 pandemic. The goal of this study was to estimate the prevalence of food insecurity in an elderly Swiss population at nutritional risk. Furthermore, the identification of risk factors and the investigation of the consequences are crucial to increase the awareness of health care workers and to take further actions. Therefore, in a second step, we aimed to find predictive factors for food insecurity and we study the influence of food insecurity regarding clinical outcomes, quality of life and health. This study may help to better identify people at risk and guide future interventions and policies.

## 2. Materials and methods

### 2.1. Study design and setting

This is a cross-sectional analysis of data from a systemic survey of participants included in the EFFORT Trial (The Effect of early nutritional support on Frailty, Functional Outcomes, and Recovery of malnourished medical inpatients Trial), a multicenter Swiss randomized controlled trial. The survey was conducted between June and August 2021 in the course of a 7-year phone call follow-up of the trial. This study included medical patients at nutritional risk hospitalized between 2014 and 2018. Nutritional risk during the initial hospital stay was defined by the Nutritional Risk Screening 2002 (NRS 2002) tool and all patients with a score ≥ 3 points (15) and with an expected length of hospital stay of more than 4 days were included. These participants were randomly assigned to receive either protocol-guided individualized nutritional support to reach protein and caloric goals (intervention group) or standard hospital food (control group). 30 days, 180 days, 3–5 years and 5–7 years after hospital discharge participants were contacted by blinded study nurses or doctoral students for structured telephone interviews. Food security was assessed in the final follow-up call 7 years after study inclusion. Detailed information about the trial have been published previously (16).

### 2.2. Assessment of food security status

We used the validated Six-Item Short Form 2012 of the U.S: Household Food Security Survey Module (17). The six-item short form of the survey module was developed by researchers at the National Center for Health Statistics of the U.S in collaboration with Abt Associates Inc. and first published in 1999 (18). The questions in the six-item module are essentially unchanged from those in the original module from 1995. There were three minor revisions in 2006, 2008, and 2012. The sum of affirmative responses to the six questions is the raw score. The food security status is assigned as follows: 0–1 points = high or marginal food security, 2–4 = low food security and 5–6 = very low food security. Further the participants can be classified into “food secure” (0–1 points) and “food insecure” (≥ 2 points). We translated the Six-Item Short Form from English to German.

### 2.3. Predictive variables

Sociodemographic characteristics (age, sex and region) were reported at study inclusion and age was extrapolated to the current date. Nutritional data (BMI, height, weight) and Barthel Index (19), living situation, education, the need of financial support and COVID-associated factors (i.e., self-reported loneliness) were assessed during the phone interview.

### 2.4. Outcome variables

Health outcomes (defined as need for rehospitalization in the last 2 years, number of hospitalizations during the last 2 years, number of falls) and quality of life [measured by EQ-5D (20)] were also structurally assessed during the telephone interview. Weight loss was calculated using the last weight from the 5-year follow-up and the current patient-reported weight. We used current BMI and weight loss data to retrospectively calculate “Malnutrition Universal Screening Tool (MUST) score (21), without considering additional scoring for acute illness, because patients were in an outpatient setting.

### 2.5. Statistical analysis

Continuous variables are expressed as mean and standard deviation (SD) and compared using student’s *t*-test, while categorical variables are shown in numbers and percentages and were analyzed by Pearson’s *χ*^2^ test. Uni- and multivariate logistic regression analyses were used to investigate possible predictive factors for food insecurity. For the analysis of associated adverse health outcomes, we used linear and logistic regression and adjusting for age. All statistical analyses were performed using Stata version 15.1 (StataCorp). *p* < 0.05 was considered statistically significant, and all tests were 2-tailed.

## 3. Results

**Figure 1:**
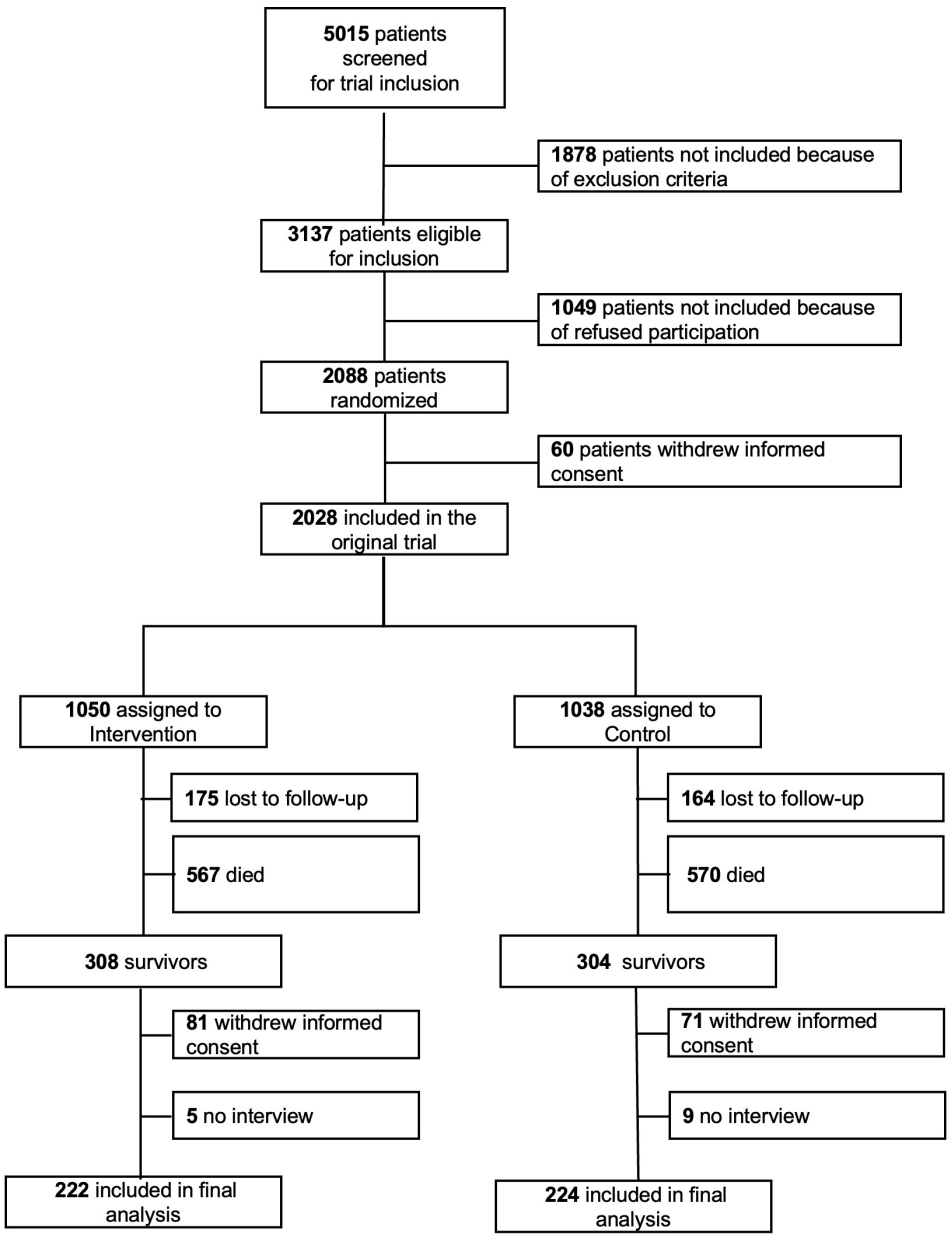
Study flow chart.

**Figure 2:**
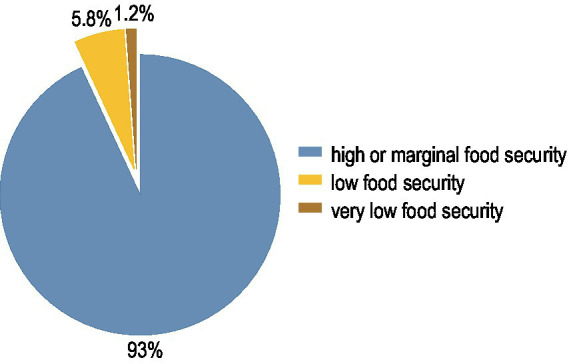
Distribution of the level of food security of all participants.

**Figure 3:**
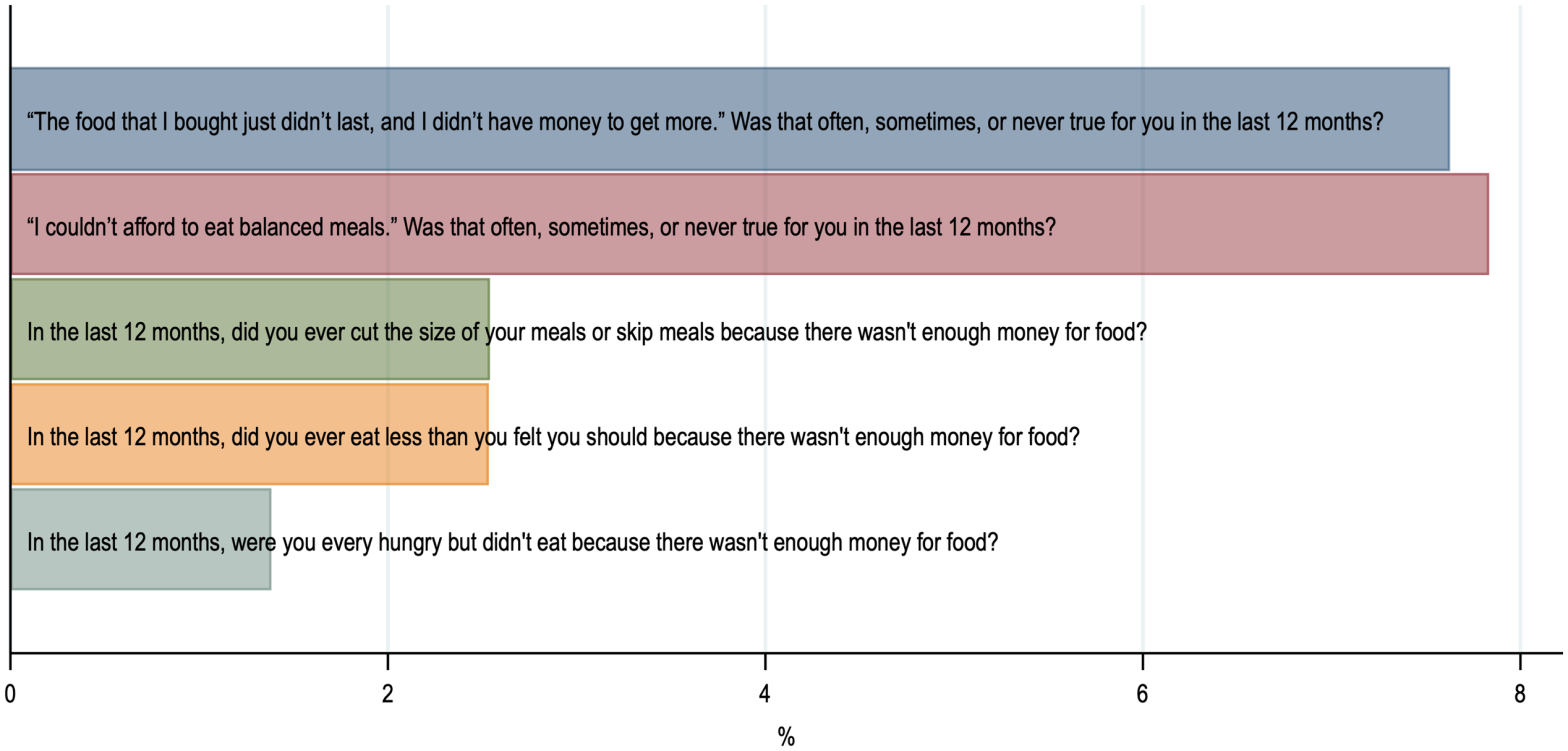
Distribution of positive answers to the questions of U.S. Household Food Security Survey Module (Six-Item Short Form).

From April 2014 to February 2018, 5,015 patients were screened and 2028 included in the initial EFFORT trial. During the 7-year follow-up 1,137 patients died and 279 were lost to follow-up. 152 patients withdrew informed consent and 27 had a missing food security questionnaire. The final analysis cohort thus consisted of 434 patients (Figure 1). Baseline characteristics for the overall population and those stratified according to food security are shown in Table 1. Of the 434 included patients, 30 (6.9%) met the definition of food insecurity. Food insecure participants were significantly younger and more independent in activities of daily living (Barthel Index). Two thirds of food insecure participants were younger than 65 years. There were no differences between male and female. Of all the food insecure participants, 50% received financial support, while only 15.8% were financially supported in the food secure group. The category food insecurity contains low (5.8%) and very low food security (1.2%), as illustrated in Figure 2. Figure 3 shows the distribution of the affirmative answers to the U.S. Household Food Security Survey Module (Six-Item Short Form).

**Table 1:** Baseline characteristics.

	Overall	Food secure	Food insecure	*p* value
*N*	434	404	30	
Sociodemographics				
Age, mean (SD)	74.18 (15.46)	75.27 (14.72)	59.60 (18.01)	<0.001
Male sex	213 (49.1%)	198 (49.0%)	15 (50.0%)	0.92
Nutritional assessment				
BMI, mean (SD)	25.65 (4.91)	25.65 (4.79)	25.63 (6.38)	0.98
Weight, mean (SD)	72.80 (16.90)	72.61 (16.29)	75.28 (23.81)	0.41
Height, mean (SD)	167.91 (9.05)	167.76 (8.94)	170.03 (10.39)	0.18
Activity of daily living				
Barthel, median (IQR)	85.91 (16.12)	85.50 (16.34)	91.50 (11.68)	0.049
Living situation				
Home without help	282 (68.8%)	263 (69.0%)	19 (65.5%)	0.75
Home with professional help	106 (25.9%)	97 (25.5%)	9 (31.0%)	
Institutionalized	22 (5.4%)	21 (5.5%)	1 (3.4%)	
Education				
Middle school	28 (6.5%)	26 (6.5%)	2 (6.7%)	0.15
High school	29 (6.7%)	24 (6.0%)	5 (16.7%)	
Apprenticeship	301 (69.8%)	280 (69.8%)	21 (70.0%)	
University	72 (16.7%)	70 (17.5%)	2 (6.7%)	
No education	1 (0.2%)	1 (0.2%)	0 (0.0%)	
Financial support	78 (18.0%)	63 (15.8%)	15 (50.0%)	<0.001
Region				
Eastern part	116 (26.7%)	108 (26.7%)	8 (26.7%)	0.94
Western part	169 (38.9%)	156 (38.6%)	13 (43.3%)	
Midlands	93 (21.4%)	87 (21.5%)	6 (20.0%)	
Central part	56 (12.9%)	53 (13.1%)	3 (10.0%)	
Loneliness				
Never	329 (76.0%)	314 (77.9%)	15 (50.0%)	<0.001
Rarely	25 (5.8%)	22 (5.5%)	3 (10.0%)	
Sometimes	43 (9.9%)	34 (8.4%)	9 (30.0%)	
Often	25 (5.8%)	24 (6.0%)	1 (3.3%)	
Always	11 (2.5%)	9 (2.2%)	2 (6.7%)	

BMI, body-mass index.

### 3.1. Predictors for food insecurity

In the univariate logistic regression analysis, we found significant associations between food insecurity and age (OR 0.95, 95% CI 0.93–0.97), governmental financial support (OR 5.35, 95% CI 2.49–11.49) and self-reported loneliness (OR 3.34 95%CI 1.54–7.26). These results remained robust in the multivariate analysis. There was no significant association between food insecurity and sex, education, housing situation, or region (Table 2).

**Table 2:** Predictors for food insecurity.

	OR univariate (95% CI)	*P* Value	OR multivariate (95% CI)	*P* Value
Sociodemographics				
Age	0.95 (0.93–0.97)	0.000	0.95 (0.92–0.98)	0.000
Male sex	1.04 (0.50–2.18)	0.917	1.67 (0.66–4.25)	0.278
Nutritional assessment				
BMI	1.00 (0.93–1.08)	0.985	0.94 (0.86–1.01)	0.104
Weight	1.01 (0.99–1.03)	0.405	–	–
Height	1.03 (0.99–1.07)	0.185	–	–
Activity of daily living				
Barthel-Index	1.03 (1.00–1.07)	0.052	1.05 (1.00–1.10)	0.072
Living situation				
Home without help	Ref		Ref	
Home with professional help	1.28 (0.56–2.94)	0.553	2.12 (0.65–6.92)	0.215
Institutionalized	0.66 (0.08–5.17)	0.692	Omitted	–
Institutionalized (y/n)	0.61 (0.08–4.72)	0.638	4.75 (0.34–65.75)	0.245
Education				
Higher education	0.51 (0.21–1.24)	0.136	0.55 (0.28–1.10)	0.093
Financial support	5.35 (2.49–11.49)	0.000	5.61 (2.11–14.86)	0.001
Region				
Ostschweiz	Ref		Ref	
Nordwestschweiz	1.13 (0.45–2.81)	0.801	1.77 (0.59–5.27)	0.305
Mittelland	0.93 (0.31–2.78)	0.898	1.04 (0.29–3.82)	0.947
Zentralschweiz	0.76 (0.19–3.00)	0.700	0.80 (0.13–4.95)	0.811
Zentralschweiz	0.76 (0.19–3.00)	0.700	0.80 (0.13–4.95)	0.811
Loneliness				
Self-reported loneliness (y/n)	3.34 (1.54–7.26)	0.002	2.71 (1.04–7.11)	0.042

### 3.2. Predictive score for food insecurity

**Figure 4:**
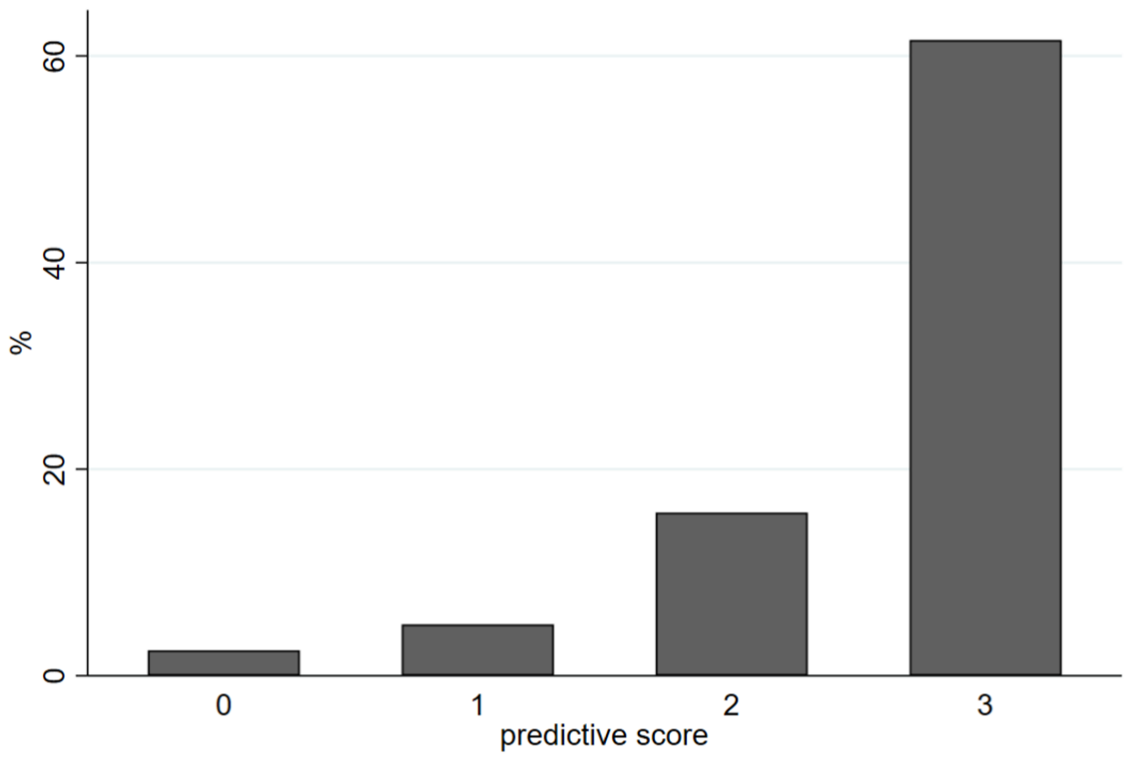
Predictive score for food insecurity containing three items: age < 65, financial support and self-reported loneliness. Each item gets one point.

We constructed a simple predictive score for food insecurity containing three items: age < 65, financial support and self-reported loneliness. We assigned one point to each of these parameters. As shown in Figure 4, the probability for food insecurity showed a stepwise increase with a low probability <5% for 0 or 1 point, to 15.8% for 2 points up to 61.5% for patients with 3 points.

### 3.3. Association of food insecurity and health, nutrition and quality of life

After adjustment for age, we found a trend toward higher risk for hospitalization in the food insecure group (OR 1.98, 95% CI 0.91–4.34), while there was no significant difference in the number of hospitalizations per person. Additionally, the odds for falls were 2.5-fold higher in the food insecure group (Table 3).

**Table 3:** Assoiciation between food insecurity and outcomes in nutrition, quality of life and health.

Outcomes	Food secure *N* or *n* (% or SD)	Food insecure *N* or *n* (% or SD)	OR or coefficient (95% CI)	value of *p*	adjusted OR or coefficient* (95% CI)	value of *p*
Health						
Hospitalization (y/n)	159 (39.4%)	15 (50.0%)	1.54 (0.73–3.24)	0.254	1.98 (0.91–4.34)	0.086
Hospitalization (n)	0.85 (1.29)	1.24 (2.01)	0.39 (−0.13–0.9)	0.138	0.43 (−0.1–0.96)*	0.110
Elective	123 (30.4%)	12 (40.0%)	1.14 (0.33–3.93)	0.841	1.28 (0.35–4.64)	0.709
Emergency	36 (8.9%)	3 (10.0%)	1.52 (0.71–3.26)	0.278	1.93 (0.86–4.29)	0.109
Falls	115 (28.7%)	10 (33.3%)	1.24 (0.56–2.74)	0.588	2.5 (1.03–6.06)	0.042
Nutrition						
Malnutrition (MUST score)	70 (17.7%)	7 (23.3%)	1.42 (0.59–3.43)	0.44	1.08 (0.43–2.72)	0.874
Weight loss last 3 months (y/n)	96 (23.9%)	9 (30.0%)	1.36 (0.6–3.07)	0.457	1.43 (0.61–3.34)	0.405
Weight change in the last 2 years in kg	−0.750 (5.52)	−0.026 (8.00)	0.72 (−1.51–2.96)	0.525	0.23 (−2.07–2.53)*	0.844
Weight loss in the last 2 years (y/n)	184 (45.5%)	7 (23.3%)	0.36 (0.15–0.87)	0.023	0.47 (0.19–1.14)	0.093
Weight loss in kg	−4.61 (4.35)	−9.81 (7.62)	−5.2 (−8.61—1.79)	0.003	−5.08 (−8.5--1.65)*	0.004
Weight gain in the last 2 years (y/n)	168 (41.6%)	19 (63.3%)	2.43 (1.13–5.23)	0.024	1.75 (0.79–3.91)	0.169
Weight gain in kg	3.95 (3.87)	4.25 (4.85)	0.3 (−1.77–2.37)	0.774	0.29 (−1.92–2.5)*	0.797
Quality of life						
Eq5d Score	0.736 (0.28)	0.68 (0.32)	−0.05 (−0.16–0.05)	0.338	−0.13 (−0.23--0.03)*	0.014
Eq5d VAS	69.45 (20.01)	61.83 (22.07)	−7.62 (−15.12--0.11)	0.047	−11.12 (−18.78--3.47)*	0.005
Barthel-Index	85.50 (16.34)	91.5 (11.68)	6 (0.03–11.98)	0.049	−1.75 (−7.24–3.75)*	0.532

BMI, body-mass index. MUST, malnutrition universal screening tool. Eq5d Score, European quality of life 5 dimensions index (EQ-5D; values range from −0·205 to 1, with higher scores indicating better quality of life). Eq5d VA, Visual-analogue scale (EQ-5D VAS; scores range from 0 to 100, with higher scores indicating better health status).

Regarding the nutritional status, there were no difference in the prevalence of malnutrition defined by the MUST score (OR 1.08, 95% CI 0.43–2.72). Total weight change did not differ between food secure and food insecure individuals. However, individually, food insecure participants showed significant higher weight loss among participants who reported weight loss (−4.62 vs. −9.81 kg, Coeff −5.2, 95% CI 8.61−1.79).

When compared to the food secure group, there was a significant lower quality of life measured by the EQ-5D index and the EQ-5D VAS when adjusted for age. There was no difference in activities of daily living and functional decline, measured by the Barthel Index and change of the Barthel Index.

## 4. Discussion

In this cross-sectional study with an elderly, multimorbid population, we found a prevalence of 6.9% for food insecurity. Significant predictors for food insecurity included lower age, need for financial support and self-reported loneliness. Food security tended to have a significant impact on health care use, falls and quality of life without directly influencing the severity of nutritional status.

In 2020, a survey done in the general Swiss population estimated the prevalence of moderate to severe food insecurity to be around 2.2% (22). That is considerably lower than the moderate to severe food insecurity in Europe and Northern America (8.8% in 2020) (1). The contrast between the prevalence of food insecurity in our study population (6.9%) and the Swiss population (2.2% in 2020) shows that our study population is an important at-risk subpopulation for food insecurity in Switzerland. Additionally, the survey was conducted in the middle of the COVID-19 pandemic. The COVID-19 pandemic triggered a global economic recession starting in 2020 and extending into 2021. The results were record levels of unemployment, lost livelihoods and rising poverty levels in many countries around the world (22) leading to rising food insecurity (10, 11).

Younger age was one of the risk factors for food insecurity, which we were able to identify. Similarly, in Canada, the prevalence of food insecurity was shown to be lower in households with seniors’ incomes as their primary income (13). This in part was interpreted in the context of Canada’s pension program, which provides some financial protection. In our population food insecurity was significant lower in the age group 65 and older (3% vs. 19.4%). We assume that similar protection mechanisms like in Canada could be in place in Switzerland through the pension program. In contrast, in Portugal and Greece data shows a trend of higher food insecurity in the elderly population (8, 23). In Greece it was estimated that 69% of older adults (≥60 years) living in the community were affected by some form of food insecurity (8). One would expect elderly residents living in an institution to be protected from food insecurity. But in our population, there were no differences in food insecurity between participants living in an institution and participants living at home.

Another significant predictor for food insecurity in our analysis was need for financial support. Of all food insecure participants, 50% received financial support and most food insecure participant, who received financial support were in working age. Similar results were found in the report “Household Food Insecurity in Canada 2021” with 63% of households relying on social assistance were food insecure (13). This raises the question, whether financial support is insufficient to prevent food insecurity. Due to the cross-sectional nature of the study, no further conclusions can be drawn. However, financial support might rather be a consequence than a cause of food insecurity.Beside age and financial support, a third predictive factor for food insecurity was self-reported loneliness. Social isolation may thus be a risk factor for food insecurity. Other studies have shown a relationship between food insecurity and poor mental health (13).

The second important question is, how does food insecurity influence clinical outcomes. In our regression analysis, there was a trend toward lower hospitalization rates and less falls in food secure patients. Previous studies showed that food insecure people are more vulnerable to chronic disease (8, 23). Chronic disease leads to higher health related expenses. And higher expenses could lead to worsening or the beginning of food insecurity. In Canada, food insecurity is associated with higher healthcare expenses (13). Scarcity in financial resources could lead to suboptimal treatment adherence, which could lead to worsening of chronic conditions, more complications and more hospitalizations. This vicious cycle could be a potential intervention point for reducing food insecurity.

Using the Malnutrition Universal Screening Tool (MUST) under the assumption that there is no acute illness present, we found no difference in the prevalence of malnutrition between food secure and food insecure participants. Other studies, which investigated the impact of food insecurity on malnutrition risk, could find an increased risk for malnutrition in food insecure participants (8, 24, 25). A study in the US showed a significant lower mean intake for 12 nutrients including energy, protein, iron, zinc, vitamins, riboflavin, niacin, B-6 and B-12 (24). An interesting finding was, that the mean weight change in the last 2 years did not differ according to food security status, but weight loss was more extreme in the food insecure individuals. In the U.S., studies have shown an association between food insecurity and risk for obesity (26), which was not found in our data.

When compared with the food secure group, there was a significant lower quality of life measured by the EQ-5D VAS and EQ-5D Score. This is congruent with what another study has shown: food insecurity having a significant impact on quality of life (23).

In previous studies, food security was associated with impaired mobility and lower activities of daily living (27). In our study activity of daily living measured by Barthel-Index was not significantly different between the two groups when adjusting for age.

### 4.1. Strengths and limitations

This is one of the first studies to report food security in a Swiss at-risk population using a standardized and validated screening tool. Additionally, the survey was conducted during a time of rising food insecurity and is therefore highly relevant. We are aware of several limitations. First, in this cross-sectional study design, outcomes happened before food security was recorded and therefore there is no proof of causality. Due to the study design, the results should be seen as hypothesis-generating and used as basis for further larger investigations in this field. Second, the population is very selected because it includes only survivors of the EFFORT study population. With our data, we can only make limited conclusions for the entire Swiss population. Because we do not have data from all regions of Switzerland, some regional differences should be expected, especially between different language regions. And it is expected that we will miss certain important subpopulations with food insecurity in our study population. For example, healthy single mothers. In the US, food insecurity is highest for single mother households and households with income below the poverty line (14). Third, we had no data on possible confounders for outcome calculation such as the presence of chronic conditions, income, household structure, smoking, alcohol consumption and health care expenses. Fourth, the approach to determine the nutrition status of participants with the MUST Score is limited. For example, protein or micronutrient deficiencies would not have shown up. And finally, we did not perform a systematic literature review and may have missed some important previous studies on the topic. As such, it is important to be able to consider all studies from different journals without delisting of specific journals (28).

### 4.2. Conclusion

In an elderly Swiss population, food insecurity was present in about 7% of the participants, particularly younger individuals with financial support, and self-reported loneliness. As food insecure individuals tended to have a higher health care use and an impaired quality of life, further scientific attention should be paid to the association between food insecurity, disease and health outcomes including strategies to improve food security status. In the assessment of malnutrition, physician and dieticians should ask for food insecurity and if detect take appropriate actions. Large, population-based assessment would be helpful to assess the prevalence and burden of food insecurity in Switzerland to understand the true magnitude of the problem.

## Data availability statement

The data analyzed in this study is subject to the following licenses/restrictions: Our analyzed data will be available to others with the publication of this manuscript on receipt of a letter of intention detailing the study hypothesis and statistical analysis plan, as already outlined in the primary EFFORT publication. Signing a data access agreement is asked from all applicants. Requests to access these datasets should be directed to schuetzph@gmail.com.

## Ethics statement

The studies involving humans were approved by the Ethics Committee of Northwest and Central Switzerland (EKNZ). The studies were conducted in accordance with the local legislation and institutional requirements. The participants provided their written informed consent to participate in this study.

## Author contributions

MR and NK-B contributed to the conception and design of the study and performed the statistical analysis. NK-B organized the database. MR wrote the first draft of the manuscript. All authors contributed to manuscript revision, read, and approved the submitted version.

## Funding

The Swiss National Science Foundation (SNSF professorship, PP00P3_150531 and PP00P3_176972) and the Research Council of the Kantonsspital Aarau, Switzerland (1410.000.058 and 1410.000.044) provided funding for the original trial.

## Conflict of interest

The authors declare that the research was conducted in the absence of any commercial or financial relationships that could be construed as a potential conflict of interest.

## Publisher’s note

All claims expressed in this article are solely those of the authors and do not necessarily represent those of their affiliated organizations, or those of the publisher, the editors and the reviewers. Any product that may be evaluated in this article, or claim that may be made by its manufacturer, is not guaranteed or endorsed by the publisher.
